# The potential protective mechanisms of melatonin on alcoholic fatty liver disease based on the animal study

**DOI:** 10.3389/fvets.2026.1827816

**Published:** 2026-06-05

**Authors:** Haixin Wu, Tianqi Zhu, Fei Guo, Lu Zhang, Bingyuan Wang, Guoshi Liu

**Affiliations:** College of Animal Science and Technology, China Agricultural University, Beijing, China

**Keywords:** apoptosis, fatty acid oxidation, hepatoprotection, lipid peroxidation, oxidative stress

## Abstract

**Introduction:**

Alcoholic fatty liver disease (AFLD) is one of the most common alcohol-related liver disorders and is characterized by hepatic lipid accumulation, oxidative stress, and hepatocyte injury. Melatonin (MT), a potent antioxidant and free-radical scavenger, has been reported to exert hepatoprotective effects; however, its protective mechanisms against chronic alcohol-induced AFLD remain incompletely understood.

**Methods:**

A mouse model of AFLD was established by chronic alcohol administration in male ICR mice. Animals were randomly assigned to a control group, an alcohol (ALC) group, and three MT treatment groups receiving 0.0025, 0.5, or 5 mg/kg/day MT. Body weight, serum AST/ALT ratio, organ coefficients, liver histopathology, hepatic lipid accumulation, malondialdehyde (MDA) levels, and the expression of oxidative stress- and apoptosis-related genes were evaluated.

**Results:**

Chronic alcohol consumption significantly reduced body weight gain, increased the serum AST/ALT ratio, induced hepatic steatosis, elevated hepatic total cholesterol (TC), triglyceride (TG), and MDA levels, and altered the expression of genes associated with oxidative stress and apoptosis. MT supplementation attenuated alcohol-induced liver injury in a dose-dependent manner. MT significantly reduced hepatic TC, TG, and MDA levels, improved liver histopathology, and suppressed alcohol-induced upregulation of Cyp2e1. In addition, MT partially modulated the expression of apoptosis-related genes, suggesting a protective role against hepatocyte damage.

**Discussion:**

Melatonin effectively alleviates chronic alcohol-induced AFLD by reducing hepatic lipid accumulation, oxidative stress, and apoptosis-related alterations. These findings provide mechanistic evidence supporting the potential application of melatonin as a complementary strategy for the prevention and management of AFLD.

## 1 Introduction

Based on the report of the World Health Organization (WHO), there are approximately 200 million individuals affected by alcohol-related disorders, among which alcoholic fatty liver disease (AFLD) is one of the main causes of liver disease-related deaths (1). AFLD is caused by long-term excessive alcohol consumption and is widely distributed worldwide. Epidemiological studies have shown that alcohol abuse is the cause of a variety of liver diseases, including hepatitis, cirrhosis, hepatocellular carcinoma, and AFLD. Unfortunately, in some developing countries, the prevalence of AFLD is increasing because of rapid economic growth accompanied by increased alcohol consumption (2).

The outcomes of AFLD are progressive and devastating. In the initial stage of AFLD, liver cells start to accumulate fat without obvious inflammation. With continued alcohol consumption, the disease progresses to alcoholic steatohepatitis (ASH), which is characterized by liver cell inflammation, damage, and dysfunction. If left untreated, chronic ASH can ultimately lead to liver fibrosis, cirrhosis, and liver failure. The mechanisms of AFLD involve multiple factors, including oxidative stress (3), hepatocyte apoptosis (4), and immune response (5). The complications of AFLD, including liver failure, portal hypertension, and liver cancer, can dramatically affect patients’ quality of life and may even be life-threatening (6).

It is well known that excessive acute alcohol consumption and long-term alcohol abuse can cause tissue oxidative stress, DNA damage, and mitochondrial dysfunction (7, 8), which ultimately lead to a variety of pathological conditions, including AFLD, alcohol-related cardiovascular dysfunction, and cognitive impairment (9). Evidence shows that even small amounts of alcohol consumption can increase reactive oxygen species (ROS) production and induce brain damage, whereas chronic alcohol consumption can cause alcoholic cardiomyopathy in rats (10, 11). Studies have shown that oxidative stress is a major contributor to AFLD, and antioxidant treatment can effectively ameliorate the disease (12).

Melatonin (MT) is a potent free-radical scavenger and antioxidant (13, 14). Not only MT but also its metabolites exhibit antioxidant capacity, which is referred to as the MT cascade reaction. It has been estimated that one MT molecule can scavenge up to 10 ROS (15). This cascade reaction makes MT more efficient as a free-radical scavenger than classical antioxidants. It can effectively protect against lipid peroxidation (16), DNA damage (17), and protein degradation (18). Its protective effects against liver injury caused by a variety of toxins have been frequently reported (19, 20), including alcohol-induced liver toxicity (21). However, few studies have systemically investigated the protective effects of MT on liver injury associated with chronic alcohol consumption. In the current study, we investigated the protective effects of MT on alcohol-induced liver damage with a focus on AFLD, through the analysis of gene expression in mice. These findings from an animal study provide novel evidence supporting MT as a potential option for the prevention and treatment of liver disorders associated with chronic alcohol consumption.

## 2 Materials and methods

### 2.1 Chemicals and agents

MT and all other chemicals and agents used in this study were purchased from Sigma-Aldrich Co. (St Louis, MO, USA) unless otherwise stated.

### 2.2 Animals

Male ICR mice aged 9–10 weeks were purchased from Charles River Company (Beijing, China). ICR mice are widely used in toxicology and pharmacology studies because of their high reproductive capacity and uniform genetic background. Male mice were selected to avoid the potential confounding effects of estrogen on alcohol metabolism and oxidative stress.

The mice were housed in a temperature-controlled room (20–22 °C) under a 12 h:12 h light/dark cycle (lights on at 6:00 a.m. and off at 6:00 p.m.) for 1 week of acclimation and were allowed free access to food and water.

All animal experiments were conducted in accordance with the guidelines of the Chinese Association for Laboratory Animal Sciences. The study protocol was approved by the Institutional Animal Care and Use Committee of China Agricultural University (protocol number of A0021306202-1-08).

### 2.3 Procedures of animal study

A total of 50 male ICR mice (30 ± 5 g) were randomly divided into five groups (*n* = 10 per group):

- Control group
- Alcohol (ALC) group (3,450 mg/kg/day ethanol by gavage)
- ALC + MT 0.0025 mg/kg/day group
- ALC + MT 0.5 mg/kg/day group
- ALC + MT 5 mg/kg/day group

MT was administered intraperitoneally (i.p.). Based on the density of ethanol, each mouse received 0.262 mL of 50% ethanol daily by gavage. Body weight was recorded once weekly.

Note: In the figures, alcohol is abbreviated as ALC.

### 2.4 Blood collection

Mice were anesthetized by intraperitoneal injection of 250 mg/kg tribromoethanol. Blood samples were collected from the orbital sinus. Serum was obtained by centrifugation at 2,000 rpm for 15 min and stored at −80 °C until analysis.

### 2.5 Tissue acquisition

After 4 or 6 weeks of treatment, animals were fasted for 24 h and euthanized by intraperitoneal injection of 400 mg/kg tribromoethanol, with reference to Siervo et al. (22).

Organs including the liver, testes, kidneys, and spleen were collected and stored at −80 °C until analysis.

### 2.6 Organ coefficient analysis

All organs were washed with normal saline (NS), dried with filter paper, and weighed individually.

The organ coefficient was calculated by dividing the weight of each organ by the body weight of the corresponding mouse.

### 2.7 Liver histological analysis

Liver tissue was fixed in 4% paraformaldehyde for 12 h and washed with distilled water. The tissues were then dehydrated through a graded ethanol series and cleared in xylene before paraffin embedding.

After cooling, tissue blocks were sectioned at a thickness of 5 μm. Sections were dewaxed with xylene, rehydrated through a graded ethanol series, and stained with hematoxylin for 10 min and eosin for 2 min.

Histological evaluation was performed under a microscope at magnifications of 20 × and 40 × .

Sample preparation was performed in the laboratory of Dr. Kuang Yu, College of Veterinary Medicine, China Agricultural University.

### 2.8 AST and ALT measurements

The liver enzymes aspartate aminotransferase (AST) and alanine aminotransferase (ALT) were measured using Mouse AST ELISA Kits and Mouse ALT ELISA Kits (Abcam, Shanghai, China) according to the manufacturer’s instructions.

The AST/ALT ratio was calculated for each mouse.

### 2.9 Biochemical analysis

Liver tissues were homogenized in ice-cold physiological saline (1:9, w/v) and centrifuged at 3,000 rpm for 15 min at 4 °C. The supernatant was collected for subsequent analyses.

Hepatic total cholesterol (TC) and triglyceride (TG) levels were measured using commercial assay kits (URIT Medical Electronics Co., Guilin, China) according to the manufacturer’s instructions. Measurements were performed using a URIT CA-431A biochemical analyzer (URIT Medical Electronics Co., Guilin, China). Results were normalized to tissue protein content and expressed as mmol/g protein.

Malondialdehyde (MDA), an indicator of lipid peroxidation, was measured using a commercial kit (Nanjing Jiancheng Bioengineering Institute, Nanjing, China) according to the manufacturer’s protocol. Absorbance was measured using an L-3180 semi-automatic biochemical analyzer (KHB, Shanghai, China). MDA levels were normalized to tissue protein content and expressed as mmol/mg protein.

### 2.10 RT-qPCR analysis

Total RNA was extracted from mouse liver using a TRIzol reagent kit (Takara Biotechnology, Dalian, China) according to the manufacturer’s instructions. The amount of RNA was determined by calculating the ratio of 260/280 nm absorbance.

cDNA was produced after reverse transcription of RNA by using the PrimeScript RT Reagent Kit (Takara Bio Inc., Tokyo, Japan). cDNA was diluted to a final concentration of 100 ng/μL for each reaction.

The amplification was performed using LightCycler® 480 II Real-Time PCR System (Roche Diagnostics, Basel, Switzerland). The template concentration was adjusted according to the kit instructions, and the expression of cytochrome P450 2E1 (*Cyp2e1*), sterol regulatory element-binding proteins (*Srebp*), and other related genes in the liver was detected by RT-qPCR.

The primers were synthesized by the Beijing Synthesis Department of Sangon Biotech (Shanghai) Co., Ltd. and are listed in Table 1.

**Table 1:** Primer sequences.

*Genes*	Primary type	Primer sequences (5′ → 3′)	Annealing temperature/°C	Production length/bp	GenBank accession no.
*β-actin*	F	CCAGCCTTCCTTCTTGGGTAT	59.4	93	NM_007393.5
	R	AGGTCTTTACGGATGTCAACG	58.0		
*Cyp2e1*	F	CGTTGCCTTGCTTGTCTGGA	63	105	NM_021282.3
	R	AAGAAAGGAATTGGGAAAGGTCC	60.4		
*Srebp*	F	GCAGCCACCATCTAGCCTG	62.5	199	NM_001313979.1
	R	CAGCAGTGAGTCTGCCTTGAT	62.1		
*Pparγ*	F	TCGCTGATGCACTGCCTATG	62.4	103	NM_001127330.2
	R	GAGAGGTCCACAGAGCTGATT	60.9		
*Bax*	F	TGAAGACAGGGGCCTTTTTG	60.2	140	NM_007527.3
	R	AATTCGCCGGAGACACTCG	62.1		
*Bcl2*	F	ATGCCTTTGTGGAACTATATGGC	60.7	120	NM_009741.5
	R	GGTATGCACCCAGAGTGATGC	62.8		
*Sirt1*	F	GCTGACGACTTCGACGACG	63	101	NM_001159589.2
	R	TCGGTCAACAGGAGGTTGTCT	62.8		
*Fas*	F	GGAGGTGGTGATAGCCGGTAT	62.9	140	NM_007988.3
	R	TGGGTAATCCATAGAGCCCAG	60.4		
*Caspase-12*	F	AGACAGAGTTAATGCAGTTTGCT	60.2	106	NM_009808.4
	R	TTCACCCCACAGATTCCTTCC	61.4		
*MnSOD*	F	CAGACCTGCCTTACGACTATGG	61.9	113	NM_013671.3
	R	CTCGGTGGCGTTGAGATTGTT	62.9		
*Pparα*	F	CCCTCGGGGAACTTAGAGGA	60.1	122	NM_011144.6
	R	CACAGAGCGCTAAGCTGTGA	60.4		
*Caspase-3*	F	AGCTTGGAACGGTACGCTAA	59.4	117	NM_001284409.1
	R	GAGTCCACTGACTTGCTCCC	60.0		
*Caspase-8*	F	GGTACTCGGCCACAGGTTAC	60.1	93	NM_009812.3
	R	TCACTGCCCAGTTCTTCAGC	60.3		
*Cyt C*	F	ACCAGCCCGGAACGAATTAAA	60.3	135	NM_007808.5
	R	CCGAACAGACCGTGGAGATT	59.8		

The PCR reaction system included 10 μL Roche quantitative mix, 0.5 μL upstream primer. and 0.5 μL downstream primer, 1 μL cDNA template, and 8 μL ddH_2_O.

The reaction procedure included pre-denaturation at 95 °C for 10 min; denaturation at 95 °C for 30 s, annealing at 60 °C for 15 s, and extension at 72 °C for 20 s, for a total of 35 cycles; followed by a final extension at 72 °C for 5 min.

The data were analyzed using the 2^-ΔΔCt^ method for relative gene expression.

### 2.11 Statistical analyses

The relative expression of target genes was calculated as 2^-ΔΔCt^. The results are expressed as mean ± standard error of the mean (SEM).

One-way analysis of variance (ANOVA) was used for data analysis, followed by a *t*-test for comparisons between groups.

A value of *p* < 0.05 was considered statistically significant.

## 3 Results

### 3.1 Effects of chronic alcohol and melatonin treatments on body weight and blood AST/ALT levels in mice

**Figure 1:**
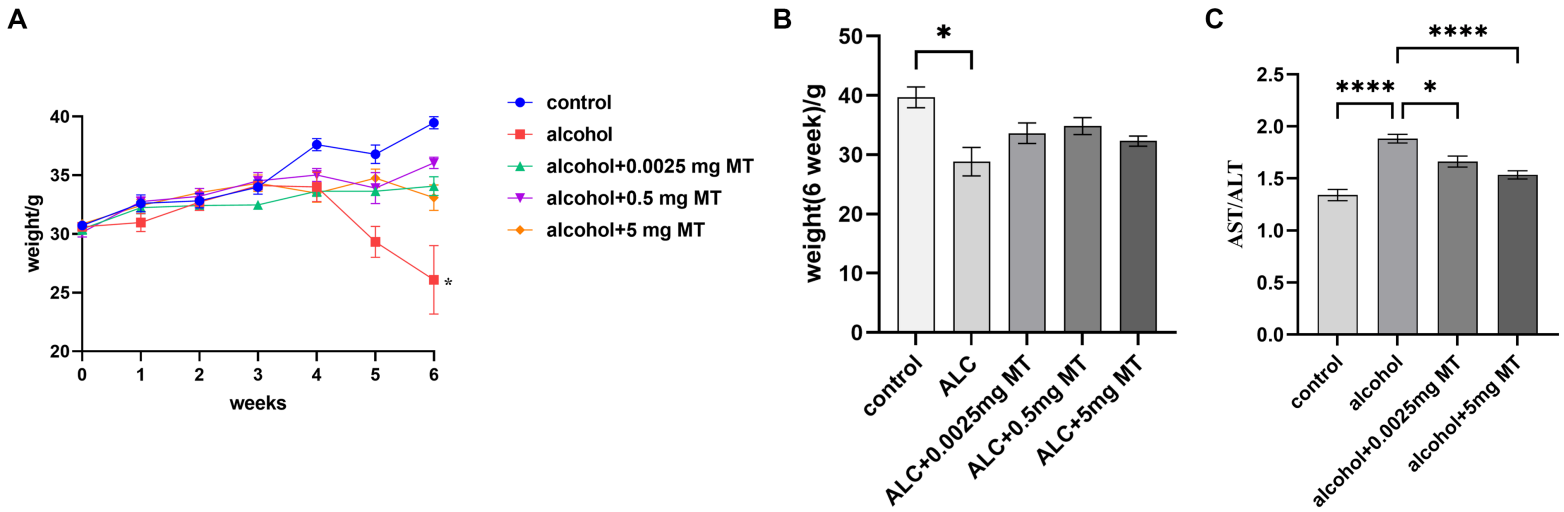
Effect of alcohol and alcohol plus melatonin treatments on body weight and the serum AST/ALT ratio in mice. **(A)** Body weight changes during chronic alcohol treatment (6 weeks) and melatonin supplementation in mice. **(B)** Average body weight after 6 weeks of treatment. **(C)** Effect of chronic alcohol treatment (6 weeks) and melatonin supplementation on the serum AST/ALT ratio in mice. Data are expressed as mean ± SEM (*n* = 10). *p < 0.05, ****p < 0.0001 versus the control group. ALT, Alanine aminotransferase; AST, aspartate aminotransferase; SEM, standard error of the mean.

The results showed that, in the initial stage, alcohol intake did not significantly impact the body weight of mice, while after 4 weeks of chronic treatment, alcohol consumption started to retard body weight gain. After 6 weeks of chronic alcohol treatment, the body weight of these mice was significantly reduced compared to the control group, whereas MT treatment at different doses significantly attenuated the body weight loss caused by chronic alcohol consumption (Figures 1A,B).

The results also showed that the ratio of serum AST/ALT in the alcohol group was significantly higher than that in the control group (*p* < 0.0001, Figure 1C), whereas MT treatment at different doses significantly reduced the AST/ALT ratio compared to the alcohol group (*p* < 0.05, Figure 1C).

### 3.2 Effect of chronic alcohol consumption and melatonin treatment on the organ coefficients of the liver and testes in mice

**Figure 2:**
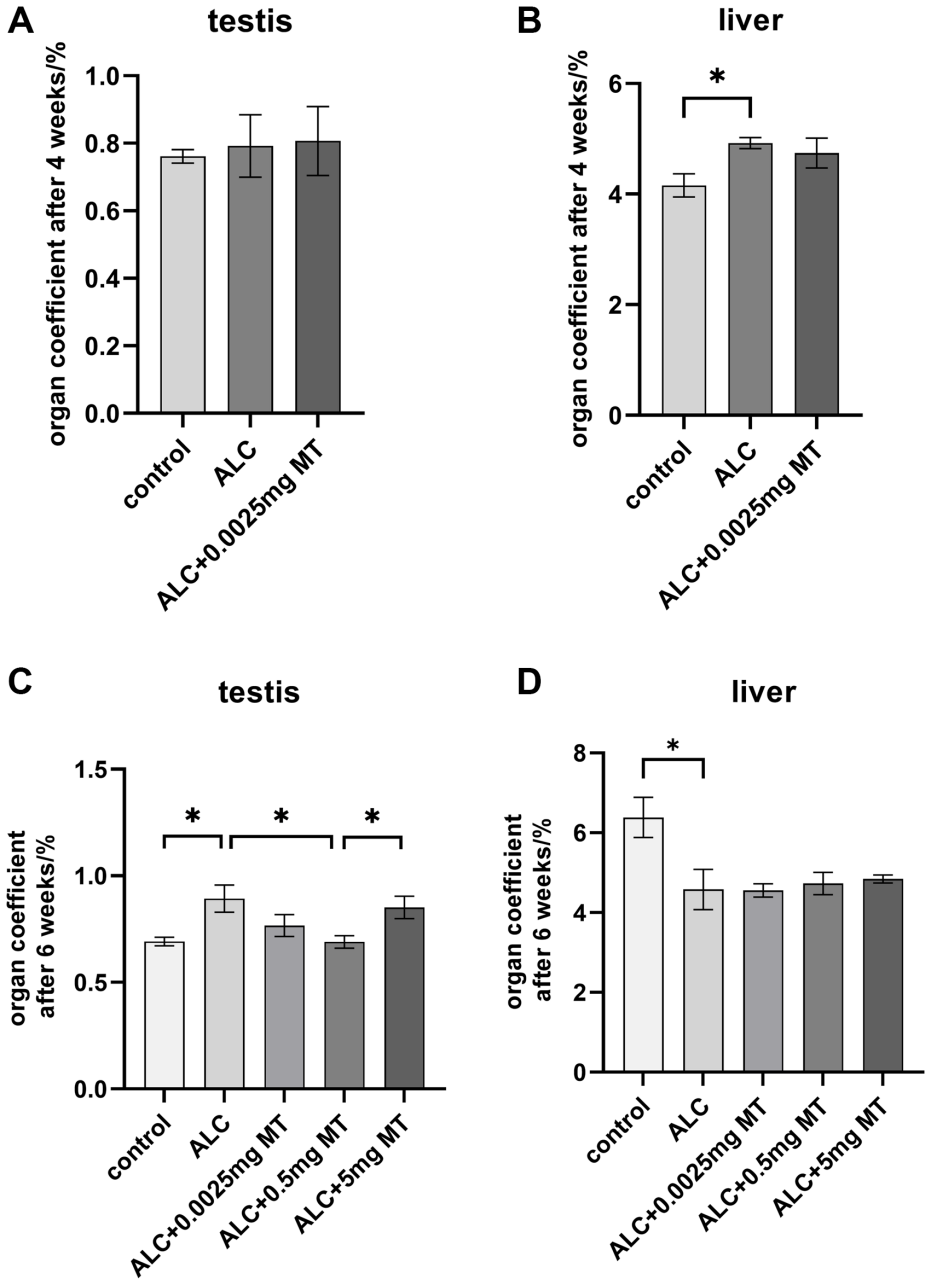
Effect of chronic alcohol consumption and melatonin treatment on the organ coefficients of the liver and testes in mice. **(A)** Testis coefficient after 4 weeks of treatment. **(B)** Liver coefficient after 4 weeks of treatment. **(C)** Organ coefficient of testis after 6 weeks of treatment. **(D)** Liver coefficient after 6 weeks of treatment. Data are expressed as mean ± SEM (*n* = 10). *p < 0.05. SEM, standard error of the mean.

The results showed that after 4 weeks of alcohol consumption, there was no significant change in testis coefficient among groups (Figure 2A), whereas the liver coefficient was significantly increased in the alcohol-treated group compared to the control group (Figure 2B) (*p* < 0.05).

However, after 6 weeks of alcohol treatment the testis coefficient was significantly increased in the alcohol-treated group compared to the control group, whereas supplementation with 0.0025 and 0.5 mg/kg MT significantly suppressed this increase (*p* < 0.05) (Figure 2C).

In contrast to the results observed after 4 weeks of alcohol treatment, 6 weeks of alcohol consumption significantly reduced the liver coefficient compared to the control group, and at this stage, MT treatment did not modify the alcohol-induced alterations liver weight (Figure 2D).

### 3.3 Effect of chronic alcohol consumption and melatonin supplementation on histopathological changes in the liver of mice

**Figure 3:**
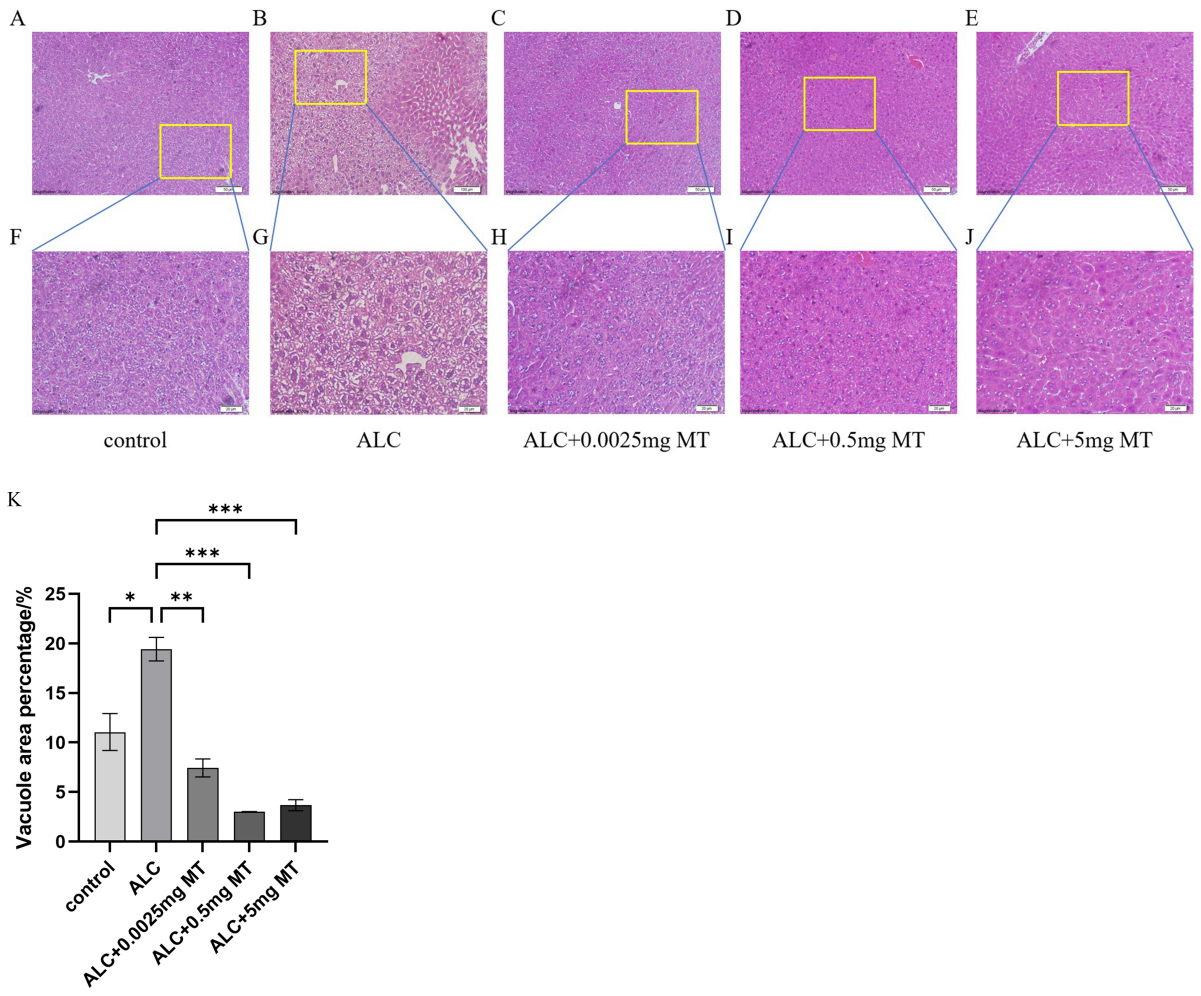
Effects of chronic alcohol treatment and melatonin supplementation on liver histomorphological changes in mice. **(A–E)** Representative histological images of mouse liver stained with H&E after 6-week treatment and captured by microscopy at 20 × magnification (scale bar = 50 μm). **(F–J)** Representative histological images of mouse liver stained with H&E after 6-week treatment and captured by microscopy under 40 × magnification (scale = 20 μm). **(K)** Quantitative analysis of vacuolar area percentage in the liver. Data are expressed as mean ± SEM (*n* = 10). *p < 0.05; **p < 0.01; ***p < 0.001.

The results of hematoxylin and eosin (H&E) staining showed normal liver histology in the control group (Figures 3A,F). After 6 weeks of chronic alcohol consumption, the liver exhibited edema and vacuolation, and the cytoplasm appeared loose, indicating alcohol-induced liver damage (Figures 3B,G). MT supplementation alleviated these histopathological alterations to varying degrees, as shown in the 0.0025 mg/kg MT group (Figures 3C,H), the 0.5 mg/kg MT group (Figures 3D,I), and the 5 mg/kg MT group (Figures 3E,J). MT supplementation significantly reduced the vacuolar area of hepatocytes (Figure 3K). Among all treatment groups, the 0.5 mg MT group exhibited the most pronounced protective effect on mouse hepatocytes (Figure 3K).

### 3.4 Effects of chronic alcohol consumption and melatonin treatment on hepatic lipid accumulation and oxidative stress in mice

**Figure 4:**
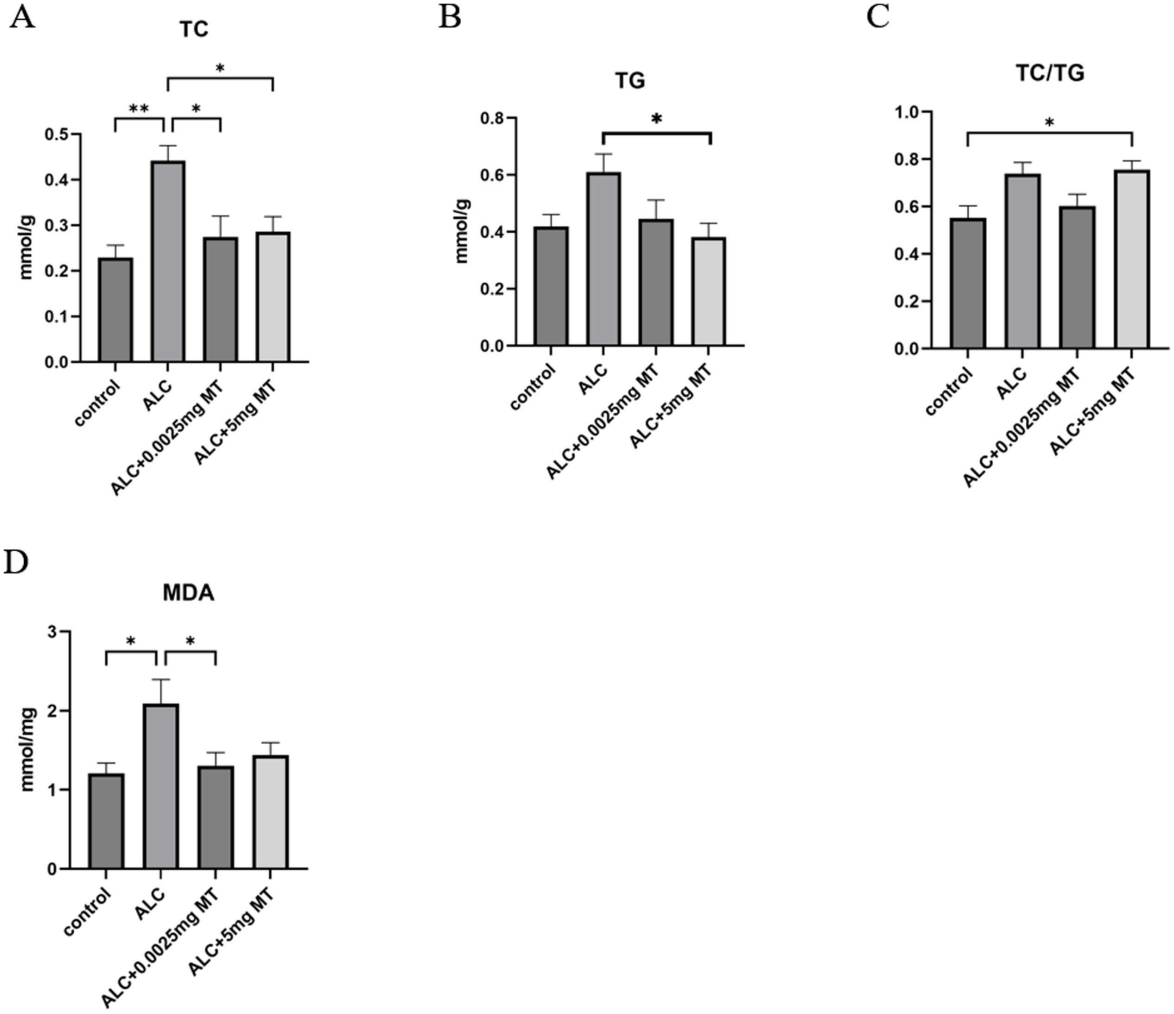
Effects of chronic alcohol treatment and melatonin supplementation on hepatic lipid accumulation and oxidative stress in mice. **(A)** Hepatic TC levels expressed as mmol/g protein. **(B)** Hepatic TG levels are expressed as mmol/g protein. **(C)** TC/TG ratio reflecting the relative composition of accumulated lipids. **(D)** Hepatic MDA levels are expressed as mmol/mg protein, a marker of lipid peroxidation. Data are expressed as mean ± SEM (*n* = 6–8). *p < 0.05; **p < 0.01. MDA, malondialdehyde; SEM, standard error of the mean; TC, total cholesterol; TG, triglyceride.

To evaluate the effects of alcohol and MT on hepatic lipid metabolism, we measured the levels of TC and TG in liver tissues. As shown in Figure 4A, chronic alcohol consumption significantly increased hepatic TC levels compared to the control group (*p* < 0.01). MT treatment at both low (0.0025 mg/kg) and high (5 mg/kg) doses significantly reduced TC levels compared to the alcohol group (*p* < 0.05 for both).

For hepatic TG levels (Figure 4B), only the high-dose MT treatment significantly reduced TG levels compared to the alcohol group (*p* < 0.05), whereas the low-dose MT showed no statistically significant effect.

The TC/TG ratio, reflecting the composition of accumulated lipids, is presented in Figure 4C. Compared to the control group, the high-dose MT group exhibited a significantly elevated TC/TG ratio (*p* < 0.05), indicating a shift in lipid composition toward relatively higher cholesterol accumulation. No significant differences in the TC/TG ratio were observed between the alcohol group and the control or MT-treated groups.

We further assessed hepatic oxidative stress by measuring MDA levels, a marker of lipid peroxidation. As shown in Figure 4D, chronic alcohol consumption markedly increased hepatic MDA content compared to the control group (*p* < 0.05). Low-dose MT treatment (0.0025 mg/kg) significantly reduced MDA levels compared to the alcohol group (*p* < 0.05), whereas high-dose MT did not show a statistically significant effect.

These results demonstrate that MT, particularly at appropriate doses, effectively alleviates alcohol-induced hepatic lipid accumulation and oxidative stress, with differential effects on TC, TG, TC/TG ratio, and MDA levels.

### 3.5 Effects of chronic alcohol consumption and melatonin treatment on the expression of oxidative stress-related genes in the liver of mice

**Figure 5:**
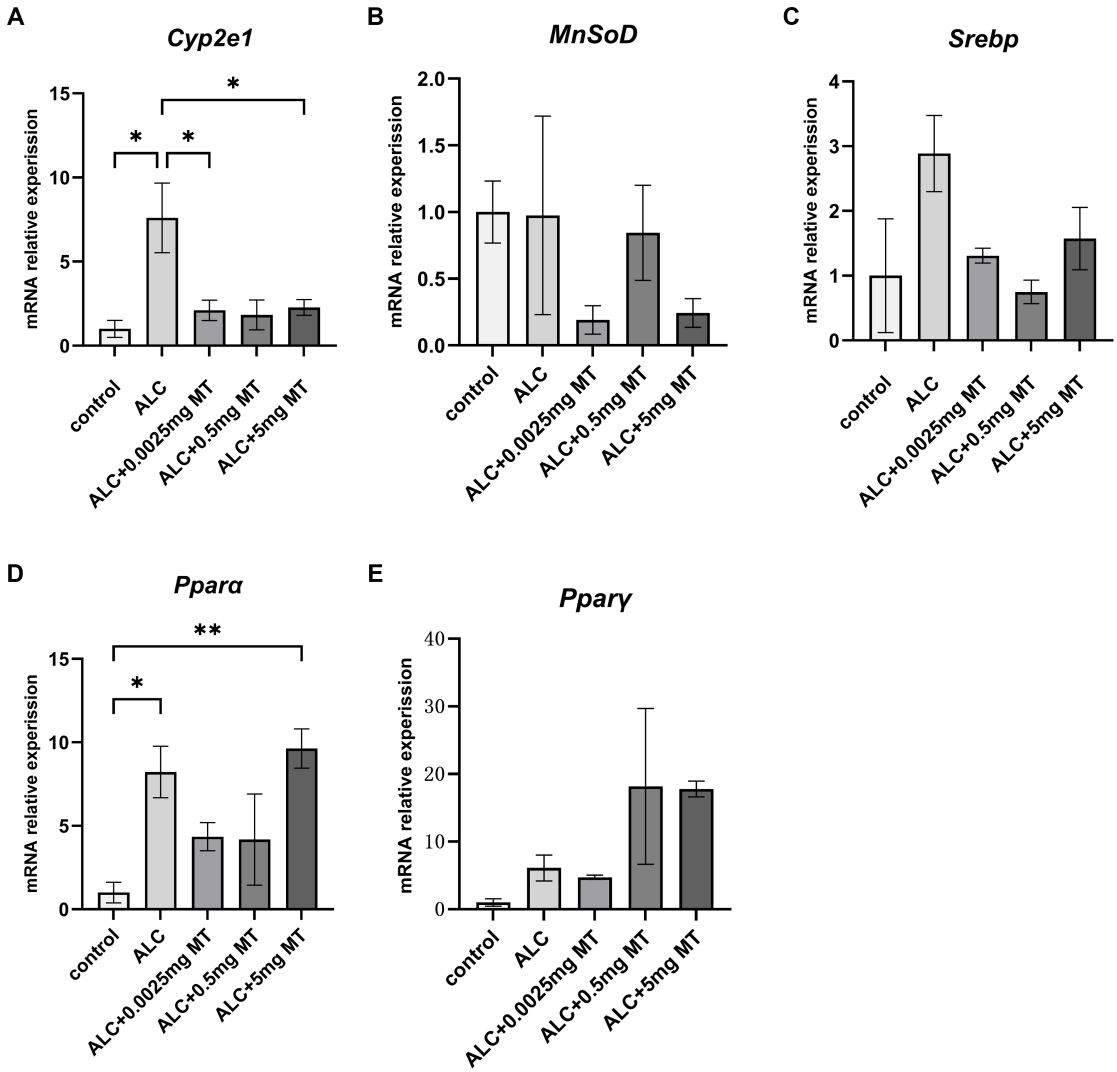
Effects of chronic (6 weeks) alcohol consumption and melatonin treatment on the expression of oxidative stress-related genes in the liver of mice. **(A)** *Cyp2e1*; **(B)** *MnSOD;* **(C)** *Srebp*; **(D)** *Pparα*; **(E)** *Pparγ*. The results were obtained by RT-qPCR analysis using *β*-actin as the reference gene. Data are expressed as mean ± SEM (*n* = 3). *p < 0.05; **p < 0.01. MnSOD, manganese superoxide dismutase; RT-qPCR, reverse transcription quantitative polymerase chain reaction; SEM, standard error of the mean.

The results showed that chronic alcohol consumption for 6 weeks significantly increased the expression of *Cyp2e1* and *Pparα* compared to the control group, whereas MT treatment significantly suppressed this increase in *Cyp2e1* expression at all doses tested (*p* < 0.05) (Figures 5A,D). No significant differences were observed in MnSOD (Figure 5B), Srebp (Figure 5C), and Pparγ (Figure 5E) among the groups.

**Figure 6:**
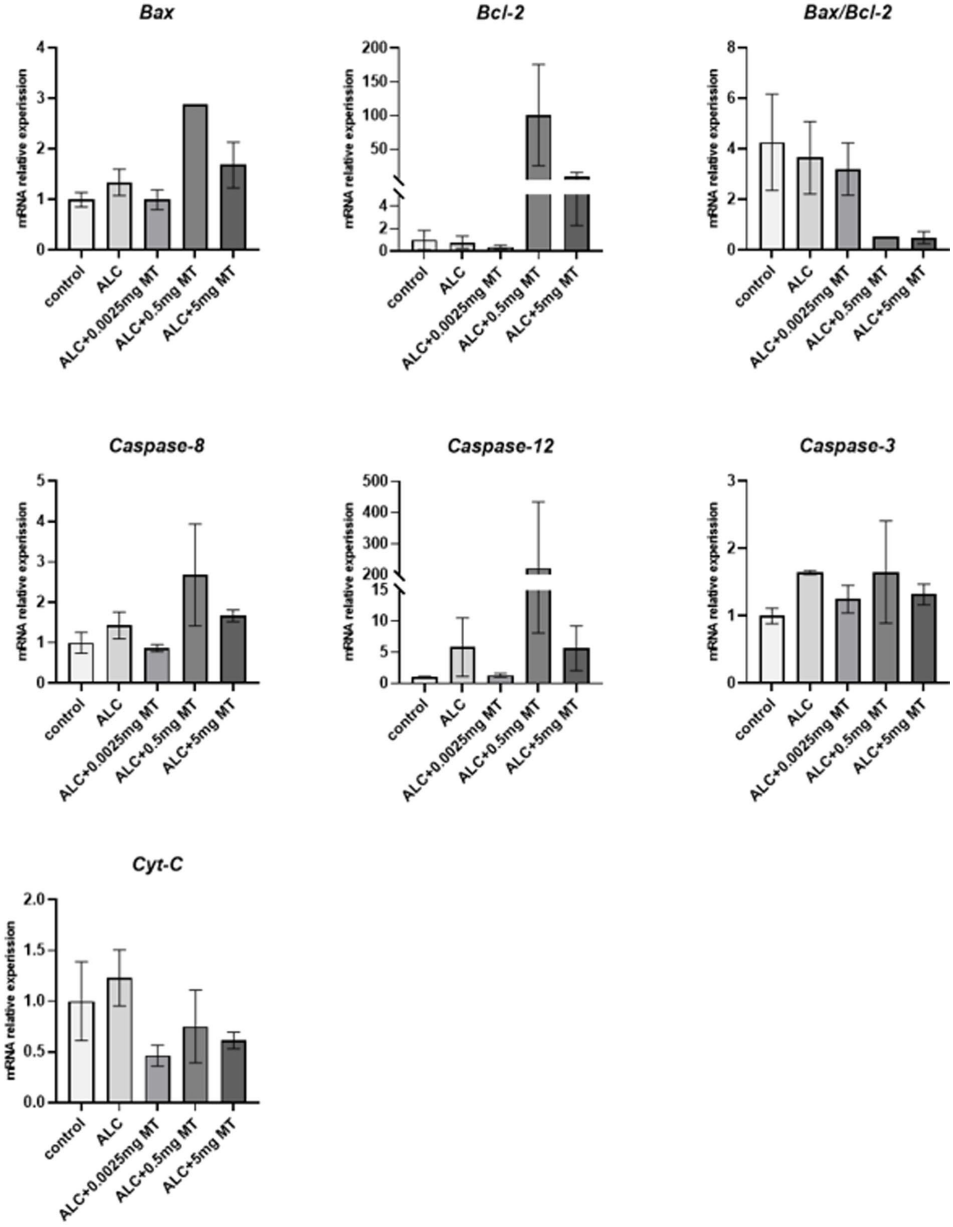
Effects of chronic (6 weeks) alcohol consumption and melatonin treatment on the expression of apoptosis-related genes in the liver of mice. **(A)** *Bax*, **(B)** *Bcl-2*, **(C)** *Bax/Bcl-2*, **(D)** *caspase-8*, **(E)** *caspase-12*, **(F)** *caspase-3*, and **(G)** C*yt-c*. The results were obtained by RT-qPCR analysis using β-actin as the reference gene. Data are expressed as mean ± SEM (*n* = 3). Cyt-c, cytochrome c; RT-qPCR, reverse transcription quantitative polymerase chain reaction; SEM, standard error of the mean.

### 3.6 Effects of chronic (6 weeks) alcohol consumption and melatonin treatment on the expression of apoptosis-related genes in the liver of mice

The results showed that the expression levels of apoptosis-related genes, including *Bax*, *Bcl-2*, *caspase-3, caspase-8, caspase-12,* and cytochrome-c (*Cyt-c*) in the alcohol-treated group did not significantly differ from those in the other groups; however, certain trends were observed. The expression level of *Caspase-3, Caspase-8, and Caspase-12* tended to increase, and MT treatment at the dose of 0.025 mg/kg tended to suppress their upregulation, whereas higher doses (0.5 and 5 mg/kg) appeared to increase their expression. Chronic alcohol treatment also showed a tendency to upregulate the *Cyt-c* expression, and MT treatment at all tested doses appeared to suppress this tendency.

## 4 Discussion

It is well documented that chronic alcohol consumption can cause a wide range of liver disorders (23). One of the most commonly encountered is AFLD, also known as steatosis. If not properly treated, the steatosis can progress to cirrhosis, liver dysfunction, and liver cancer (24). The histological appearance of AFLD is characterized by the accumulation of phospholipids, cholesterol and TGs in the liver, forming lipid droplets in hepatocytes around the central veins and then progressively developing into hepatitis in the middle of the lobules and around the portal veins (25). The underlying mechanisms of AFLD are multifactorial; however, alcohol metabolism–induced alterations in gene expression play a critical role in its development.

Consistent with the biochemical measurements, our data showed that chronic alcohol consumption significantly elevated hepatic TC and MDA levels, indicating alcohol-induced lipid accumulation and oxidative stress. MT treatment at both low and high doses effectively reduced TC levels, whereas only the high dose significantly lowered TG levels, suggesting that the two lipids respond differently to MT intervention. Notably, the TC/TG ratio was significantly increased only in the high-dose MT group compared to the control group, implying that high-dose MT may preferentially suppress TG synthesis or enhance TG export, leading to a relative increase in cholesterol proportion. The ability of MT to modulate *β*-oxidation and mitochondrial function, as previously demonstrated in prediabetic rats (26), may partly explain this dose-dependent effect. Furthermore, the protective effect of low-dose MT against alcohol-induced oxidative stress, evidenced by the significant reduction in hepatic MDA levels, aligns with the well-established antioxidant properties of MT. Multiple studies have confirmed that MT effectively suppresses lipid peroxidation and reduces MDA accumulation in ethanol-exposed animals (27, 28). MT has also been shown to prevent AFLD through its actions on the gut-liver axis, mitigating endotoxemia and intestinal barrier dysfunction (29). Collectively, these findings highlight the importance of dose optimization when considering MT as a therapeutic agent for AFLD, as low and high doses may exert distinct modulatory effects on lipid metabolism and oxidative stress (30).

The liver is the main organ for alcohol metabolism in mammals, with *Cyp2e1* being the primary enzyme involved in alcohol metabolism. The activation of *Cyp2e1*, as well as NADPH, during alcohol metabolism promotes ROS generation and hepatocyte oxidative stress, which leads to liver injury (31, 32). *Cyp2e1*-mediated oxidative stress then inhibits hepatic fatty acid oxidation. In addition, oxidative stress induced by chronic alcohol consumption also upregulates the expression of *Srebp*, which in turn upregulates lipid synthesis genes and promotes the accumulation of TGs in hepatocytes (33). The inhibition of fatty acid oxidation and increased lipid synthesis caused by alcohol metabolism contribute to AFLD.

In the current study, we confirmed that chronic alcohol treatment induces AFLD in mice. This was evidenced by the increased AST/ALT ratio (an indication of liver damage), lipid accumulation in hepatocytes, and altered expression of oxidative stress-related and apoptosis-related genes, particularly the upregulation of *Cyp2e1* and *Pparα*. However, these alterations induced by chronic alcohol consumption were partially or completely reversed by MT treatment. As an antioxidant, MT has been used in a variety of oxidative stress-associated liver disorders, including diabetes (30), hyperlipidemia (34), and AFLD (35).

MT supplementation also had a positive effect on alleviating alcohol-induced apoptosis. The ability of MT to downregulate alcohol-induced *Cyp2e1* expression and suppress ROS production and liver injury is probably mediated through targeting *Sirt1*, as silencing of *Sirt1* significantly limits these beneficial effects of MT (36). *Bcl2* and *Bax* are important regulators of apoptosis, with *Bax* acting as a pro-apoptotic gene and *Bcl2* acting as an anti-apoptotic gene (37). In this study, MT at certain doses exhibited a tendency to lower the *Bax/Bcl2* expression ratio in the mouse liver, suggesting that MT may have the capacity to reduce liver cell apoptosis induced by chronic alcohol consumption, which is consistent with the histological findings.

In alcoholic liver disease, endoplasmic reticulum stress leads to the activation of *Caspase-12* (38), whereas alcohol promotes Kupffer cells to release tumor necrosis factor-*α* and activate *caspase-8* (39). Ultimately, *caspase-3* activation leads to hepatocyte apoptosis through the combined action of these two pathways (40). In this study, following chronic alcohol treatment, *caspase-3*, *caspase-8*, and *caspase-12* all showed an upward trend in expression, and this abnormal trend was alleviated by low-dose MT supplementation.

*Cyt-c* is located in the inner mitochondrial membrane (41). *Cyt-c* released from mitochondria is a key signaling molecule that initiates the intrinsic apoptotic pathway, leading to programmed cell death through activation of the caspase cascade (42). Meanwhile, *Cyt-c* also regulates apoptosis through the regulation of ROS induced by endoplasmic reticulum stress (43). After alcohol treatment, *Cyt-c* expression in mouse liver showed an increasing trend, suggesting enhanced mitochondrial-mediated hepatocyte apoptosis. MT supplementation at all doses used in this study reduced *Cyt-c* expression and alleviated the increase in liver cell damage, as indicated by the histological observations.

The compelling protective effects of MT on AFLD observed in our murine model naturally prompt consideration of its therapeutic potential in human AFLD. Our data demonstrate that MT effectively mitigates AFLD by reducing lipid accumulation, oxidative stress, and apoptosis in a mouse model. The results provide mechanistic support for the use of MT as a supplemental strategy for high-risk individuals to prevent or delay the onset of AFLD, as MT has few or no serious side effects (44).

We acknowledge several limitations of this study. The relatively small sample size in each experimental group may limit the generalizability of our findings and reduce the statistical power to detect more subtle phenotypic changes. However, the effects of alcohol treatment on mouse liver morphology and function, as well as the beneficial effects of MT supplementation on AFLD, are consistent with previous reports. In addition, although H&E staining clearly demonstrated alcohol-induced hepatic steatosis, we did not perform Masson staining for fibrosis assessment because significant fibrosis is not typically expected in a 6-week alcohol-feeding model. Oxidative stress was assessed by measuring MDA levels and the expression of antioxidant-related genes (*Cyp2e1* and *MnSOD*), which are well-established markers. Future studies with larger sample sizes are warranted to confirm these findings and explore additional subtle effects.

In conclusion, this study demonstrates that MT ameliorates alcohol-induced fatty liver disease in a dose-dependent manner by regulating hepatic lipid accumulation and oxidative stress. Chronic alcohol consumption significantly increased hepatic TC and MDA levels, whereas MT treatment effectively reversed these alterations. Notably, low-dose MT (0.0025 mg/kg) significantly reduced both TC and MDA levels, whereas high-dose MT (5 mg/kg) preferentially lowered TG levels and increased the TC/TG ratio, suggesting a dose-dependent shift in hepatic lipid composition. At the molecular level, MT downregulated alcohol-induced *Cyp2e1* expression, contributing to reduced oxidative stress. These findings provide mechanistic support for the potential use of MT as a complementary strategy for preventing or delaying AFLD, with dose optimization representing an important consideration for future therapeutic applications.

## Data Availability

The original contributions presented in the study are included in the article/supplementary material, further inquiries can be directed to the corresponding authors.

## References

[1] World Health Organization. Global Status Report on Alcohol 2004. 2nd ed. Geneva, Switzerland: World Health Organization (2004).

[2] Gonzalez-Chagolla A Olivas-Martinez A Ruiz-Manriquez J Servín-Rojas M Kauffman-Ortega E Chávez-García LC . Cirrhosis etiology trends in developing countries: transition from infectious to metabolic conditions. Report from a multicentric cohort in Central Mexico. Lancet Reg Health Am. (2021) 7:100151. doi: 10.1016/j.lana.2021.100151, 36777654 PMC9904121

[3] LeFort KR Rungratanawanich W Song BJ. Contributing roles of mitochondrial dysfunction and hepatocyte apoptosis in liver diseases through oxidative stress, post-translational modifications, inflammation, and intestinal barrier dysfunction. Cell Mol Life Sci. (2024) 81:34. doi: 10.1007/s00018-023-05061-7, 38214802 PMC10786752

[4] Heo MJ Kim TH You JS Blaya D Sancho-Bru P Kim SG. Alcohol dysregulates miR-148a in hepatocytes through FoxO1, facilitating pyroptosis via TXNIP overexpression. Gut. (2019) 68:708–20. doi: 10.1136/gutjnl-2017-315123, 29475852 PMC6581021

[5] Wu X Fan X McMullen MR Miyata T Kim A Pathak V . Macrophage-derived MLKL in alcohol-associated liver disease: regulation of phagocytosis. Hepatology. (2023) 77:902–19. doi: 10.1002/hep.32612, 35689613 PMC9741663

[6] Jophlin LL Singal AK Bataller R Wong RJ Sauer BG Terrault NA . ACG clinical guideline: alcohol-associated liver disease. Am J Gastroenterol. (2024) 119:30–54. doi: 10.14309/ajg.0000000000002572, 38174913 PMC11040545

[7] Matharu Z Enomoto J Revzin A. Miniature enzyme-based electrodes for detection of hydrogen peroxide release from alcohol-injured hepatocytes. Anal Chem. (2013) 85:932–9. doi: 10.1021/ac3025619, 23163580

[8] Goldman A Chen HDR Roesly HB Hill KA Tome ME Dvorak B . Characterization of squamous esophageal cells resistant to bile acids at acidic pH: implication for Barrett's esophagus pathogenesis. Am J Physiol Gastrointest Liver Physiol. (2011) 300:G292–302. doi: 10.1152/ajpgi.00461.2010, 21127259 PMC3043651

[9] Letenneur L. Risk of dementia and alcohol and wine consumption: a review of recent results. Biol Res. (2004) 37:189–93. doi: 10.4067/S0716-97602004000200003, 15455646

[10] Wang X Zhou J Guo X Su H. Blood rheology in patients with malignant tumors with distant metastases. Chin J Hemorheol. (2002) 12:238.

[11] Mchedlishvili G Shakarishvili R Momtselidze N Gobejishvili L Aloeva M Mantskava M. Comparative values of erythrocyte aggregability versus other indices of hemorheological disorders in patients with ischemic brain infarcts. Clin Hemorheol Microcirc. (2000) 22:9–15. 10711816

[12] Cichoż-Lach H Michalak A. Oxidative stress as a crucial factor in liver diseases. World J Gastroenterol. (2014) 20:8082–91. doi: 10.3748/wjg.v20.i25.8082, 25009380 PMC4081679

[13] Pieri C Marra M Moroni F Recchioni R Marcheselli F. Melatonin: a peroxyl radical scavenger more effective than vitamin E. Life Sci. (1994) 55:PL271–6. doi: 10.1016/0024-3205(94)00666-0, 7934611

[14] Metzger BE Coustan DR. Summary and recommendations of the fourth international workshop-conference on gestational diabetes mellitus. The organizing committee. Diabetes Care. (1998) 21 Suppl 2:B161–7. 9704245

[15] Tan DX Manchester LC Esteban-Zubero E Zhou Z Reiter RJ. Melatonin as a potent and inducible endogenous antioxidant: synthesis and metabolism. Molecules. (2015) 20:18886–906. doi: 10.3390/molecules201018886, 26501252 PMC6332205

[16] Vural H Sabuncu T Arslan SO Aksoy N. Melatonin inhibits lipid peroxidation and stimulates the antioxidant status of diabetic rats. J Pineal Res. (2001) 31:193–8. doi: 10.1034/j.1600-079X.2001.310301.x, 11589752

[17] Xu D Liu L Zhao Y Yang L Cheng J Hua R . Melatonin protects mouse testes from palmitic acid-induced lipotoxicity by attenuating oxidative stress and DNA damage in a SIRT1-dependent manner. J Pineal Res. (2020) 69:e12690. doi: 10.1111/jpi.12690, 32761924

[18] Kim CH Kim KH Yoo YM. Melatonin-induced autophagy is associated with degradation of MyoD protein in C2C12 myoblast cells. J Pineal Res. (2012) 53:289–97. doi: 10.1111/j.1600-079X.2012.00998.x, 22582971

[19] Liu S Kang W Mao X Ge L Du H Li J . Melatonin mitigates aflatoxin B1-induced liver injury via modulation of gut microbiota/intestinal FXR/liver TLR4 signaling axis in mice. J Pineal Res. (2022) 73:e12812. doi: 10.1111/jpi.12812, 35652241

[20] Zhu L Zhang Q Hua C Ci X. Melatonin alleviates particulate matter-induced liver fibrosis by inhibiting ROS-mediated mitophagy and inflammation via Nrf2 activation. Ecotoxicol Environ Saf. (2023) 268:115717. doi: 10.1016/j.ecoenv.2023.115717, 37992643

[21] Sousa Coelho IDD Lapa Neto CJC Souza TGDS Silva MAD Chagas CA Santos KRPD . Protective effect of exogenous melatonin in rats and their offspring on the genotoxic response induced by the chronic consumption of alcohol during pregnancy. Mutat Res Genet Toxicol Environ Mutagen. (2018) 832-833:52–60. doi: 10.1016/j.mrgentox.2018.06.018, 30057021

[22] Siervo GE Vieira HR Ogo FM Fernandez CD Gonçalves GD Mesquita SF . Spermatic and testicular damages in rats exposed to ethanol: influence of lipid peroxidation but not testosterone. Toxicology. (2015) 330:1–8. doi: 10.1016/j.tox.2015.01.016, 25637669

[23] Israelsen M Francque S Tsochatzis EA Krag A. Steatotic liver disease. Lancet. (2024) 404:1761–78. doi: 10.1016/S0140-6736(24)01811-7, 39488409

[24] Blachier M Leleu H Peck-Radosavljevic M Valla DC Roudot-Thoraval F. The burden of liver disease in Europe: a review of available epidemiological data. J Hepatol. (2013) 58:593–608. doi: 10.1016/j.jhep.2012.12.005, 23419824

[25] Gao B Bataller R. Alcoholic liver disease: pathogenesis and new therapeutic targets. Gastroenterology. (2011) 141:1572–85. doi: 10.1053/j.gastro.2011.09.002, 21920463 PMC3214974

[26] Peter Guengerich F Avadhani NG. Roles of cytochrome P450 in metabolism of ethanol and carcinogens. Adv Exp Med Biol. (2018) 1032:15–35. doi: 10.1007/978-3-319-98788-0_2, 30362088 PMC6371814

[27] Kim MJ Sim MO Lee HI Ham JR Seo KI Lee MK. Dietary umbelliferone attenuates alcohol-induced fatty liver via regulation of PPARα and SREBP-1c in rats. Alcohol. (2014) 48:707–15. doi: 10.1016/j.alcohol.2014.08.008, 25262573

[28] Delpino FM Figueiredo LM Nunes BP. Effects of melatonin supplementation on diabetes: a systematic review and meta-analysis of randomized clinical trials. Clin Nutr. (2021) 40:4595–605. doi: 10.1016/j.clnu.2021.06.007, 34229264

[29] Mohammadi-Sartang M Ghorbani M Mazloom Z. Effects of melatonin supplementation on blood lipid concentrations: a systematic review and meta-analysis of randomized controlled trials. Clin Nutr. (2018) 37:1943–54. doi: 10.1016/j.clnu.2017.11.003, 29191493

[30] LeFort KR Rungratanawanich W Song BJ. Melatonin prevents alcohol- and metabolic dysfunction- associated steatotic liver disease by mitigating gut dysbiosis, intestinal barrier dysfunction, and endotoxemia. Antioxidants. (2023) 13:43. doi: 10.3390/antiox13010043, 38247468 PMC10812487

[31] de Souza MC Agneis MLG das Neves KA de Almeida MR Feltran GDS Souza Cruz EM . Melatonin improves lipid homeostasis, mitochondrial biogenesis, and antioxidant defenses in the liver of prediabetic rats. Int J Mol Sci. (2025) 26:4652. doi: 10.3390/ijms26104652, 40429795 PMC12111231

[32] Kurhaluk N Sliuta A Kyriienko S Winklewski PJ. Melatonin restores white blood cell count, diminishes glycated haemoglobin level and prevents liver, kidney and muscle oxidative stress in mice exposed to acute ethanol intoxication. Alcohol Alcohol. (2017) 52:521–8. doi: 10.1093/alcalc/agx045, 28854709

[33] Esrefoglu M Cetin A Taslidere E Elbe H Ates B Tok OE . Therapeutic effects of melatonin and quercetin in improvement of hepatic steatosis in rats through supression of oxidative damage. Bratisl Lek Listy. (2017) 118:347–54. doi: 10.4149/BLL_2017_066, 28664744

[34] Zhang JJ Meng X Li Y Zhou Y Xu DP Li S . Effects of melatonin on liver injuries and diseases. Int J Mol Sci. (2017) 18:673. doi: 10.3390/ijms18040673, 28333073 PMC5412268

[35] Kromm F Baumann A Sánchez V Brandt A Staltner R Bergheim I . Oral supplementation of melatonin attenuates the onset of alcohol-related liver disease. J Mol Med (Berl). (2025) 103:1219–1230. doi: 10.1007/s00109-025-02583-4, 40775550 PMC12449381

[36] Lee SE Koh H Joo DJ Nedumaran B Jeon HJ Park CS . Induction of SIRT1 by melatonin improves alcohol-mediated oxidative liver injury by disrupting the CRBN-YY1-CYP2E1 signaling pathway. J Pineal Res. (2020) 68:e12638. doi: 10.1111/jpi.12638, 32053237

[37] Hanahan D Weinberg RA. Hallmarks of cancer: the next generation. Cell. (2011) 144:646–74. doi: 10.1016/j.cell.2011.02.013, 21376230

[38] Jiang S Xie Q Zhou H Zhang W Zhou X Li G . Ribozyme-mediated inhibition of caspase-12 activity reduces apoptosis induced by endoplasmic reticulum stress in primary mouse hepatocytes. Int J Mol Med. (2008) 22:717–24. 19020768

[39] Song Z Zhou Z Uriarte S Wang L Kang YJ Chen T . S-adenosylhomocysteine sensitizes to TNF-alpha hepatotoxicity in mice and liver cells: a possible etiological factor in alcoholic liver disease. Hepatology. (2004) 40:989–97. doi: 10.1002/hep.20412, 15382170

[40] Sui B Wang R Chen C Kou X Wu D Fu Y . Apoptotic vesicular metabolism contributes to organelle assembly and safeguards liver homeostasis and regeneration. Gastroenterology. (2024) 167:343–56. doi: 10.1053/j.gastro.2024.02.001, 38342194

[41] Garrido C Galluzzi L Brunet M Puig PE Didelot C Kroemer G. Mechanisms of cytochrome c release from mitochondria. Cell Death Differ. (2006) 13:1423–33. doi: 10.1038/sj.cdd.4401950 16676004

[42] Morse PT Pasupathi V Vuljaj S Yazdi N Zurek MP Wan J . Cardiac tyrosine 97 phosphorylation of cytochrome *c* regulates respiration and apoptosis. Int J Mol Sci. (2025) 26:1314. doi: 10.3390/ijms26031314, 39941082 PMC11818311

[43] Xie H Song L Katz S Zhu J Liu Y Tang J . Electron transfer between cytochrome c and microsomal monooxygenase generates reactive oxygen species that accelerates apoptosis. Redox Biol. (2022) 53:102340. doi: 10.1016/j.redox.2022.102340, 35609401 PMC9130584

[44] Besag FMC Vasey MJ. Adverse events in long-term studies of exogenous melatonin. Expert Opin Drug Saf. (2022) 21:1469–81. doi: 10.1080/14740338.2022.2160444, 36562403

